# Balancing the Risks and Benefits of COVID-19 Vaccination for Pregnant Women and Their Children

**DOI:** 10.3389/fimmu.2021.748456

**Published:** 2021-12-16

**Authors:** Chengliang Yang, Hedi Zhao, Scott J. Tebbutt

**Affiliations:** ^1^ Prevention of Organ Failure (PROOF) Centre of Excellence and Centre for Heart Lung Innovation, St Paul’s Hospital, University of British Columbia, Vancouver, BC, Canada; ^2^ Faculty of Medicine, McGill University, Montreal, QC, Canada; ^3^ Division of Respiratory Medicine, Department of Medicine, University of British Columbia, Vancouver, BC, Canada

**Keywords:** COVID-19, SARS-CoV-2, vaccine, pregnant women, newborns

Approximately 140 million babies were born worldwide in 2020, yet nearly all coronavirus disease 2019 (COVID-19) vaccine clinical trials have excluded pregnant and breastfeeding individuals from participating. In the meantime, pregnant women and their clinicians have little data to make informed decisions on immunization safety. With the continued global escalation of COVID-19 cases and studies demonstrating greater morbidity from COVID-19 in pregnant women (1), there is an urgent need for guidance to assist health care providers in performing individualized risk assessments with pregnant and breastfeeding people. Though it is reasonable to suggest that pregnant and breastfeeding individuals receive the COVID-19 vaccine, its safety in this population has yet to be elucidated. We raise a number of concerns in this article.

A recent report of 35,691 pregnant women who received the mRNA COVID-19 vaccination provided support for its feasibility though the data was insufficient to draw conclusions (2). Ideally, the data should be presented with reference to baseline data on hypertension, obesity, or diabetes, all of which are risk factors implicated both in pregnancy and COVID-19 (3, 4). Incidence of maternal fever and associated acetaminophen use should also be considered given their association with birth defects and neurodevelopmental and behavioral disorders such as attention deficit hyperactivity disorder (ADHD), respectively (5–10). Until more research is conducted, the BNT162b2 vaccine (Pfizer-BioNTech-vaccine) may be more appropriate during pregnancy than the mRNA-1273 vaccine (Moderna vaccine) for its lower rate of fever (24.8% vs 46.0%) (2).

Similarly, in newborns there is a paucity of data on any benefit of the mRNA COVID-19 vaccine to draw definitive conclusions. In a small study, Collier and colleagues reported that receipt of the mRNA COVID-19 vaccine was immunogenic in nine participants, and vaccine-elicited antibodies could be identified in infant cord blood (11). These antibodies have also been identified in breast milk, though enzymatic digestion is likely to eliminate any biological efficacy (12). Nevertheless, in the absence of effective humoral immunity following birth, passive immunity is essential and will likely play a vital role in newborn and infant health outcomes in future pandemics. Following this vulnerable period, active immunity can be acquired through infection, illness or vaccination. It is uncertain whether this trained immunity is effective as studies have shown declining immunity around six to nine months after vaccination (13). Of concern, children aged less than 2 years who contract COVID-19 are more likely to be hospitalized than older children (14). Thus, we encourage investigators to continue to investigate vaccine efficacy in newborns, track maternal prognoses and the impact of vaccines on infant development and evaluate whether vaccinated pregnant individuals confer protection to their offspring *via* the placenta or human milk. As reported by The Council for International Organizations of Medical Sciences (CIOMS), the inclusion of pregnant women and breastfeeding women in COVID-19 vaccine trials are urgently needed to identify the potential risks and benefits in this population, as well as to the fetus or nursing infant.

Some Australian clinicians have recommended that women undergoing *in vitro* fertilization (IVF) treatment should avoid vaccinating in the days leading up to their embryo transfer or egg collection^1^. Indeed, the stride towards vaccination should be met with prudence, as safety data is limited and health agency guidelines are vague and, in some cases, controversial (15). Importantly, the COVID-19 vaccines have not been specifically evaluated for pregnant individuals utilizing IVF. A pivotal addition to ongoing studies would be to incorporate an analysis of the pregnancy and infant outcomes in individuals utilizing IVF and how these contrast to the established cumulative live-birth rate of 51% following six cycles of IVF (16).

Lastly, the short-term and long-term effects of COVID-19 vaccination in pregnant and lactating women, the fetus, and infants remain unanswered. Since the immune response to COVID-19 vaccination in pregnant and breastfeeding women cannot be inferred from that of non-pregnant and non-breastfeeding women, we must highlight the importance of enrolling these individuals in clinical trials. The long-term safety data that speak most clearly to the question of whether the COVID-19 vaccines benefit pregnant women and newborns should be obtained from robust clinical trials. Emerging evidence suggests that global maternal and fetal outcomes have worsened during the pandemic, with a significant increase in maternal and fetal mortality (particularly in low-income and middle-income countries), ruptured ectopic pregnancies, and maternal symptoms of depression (17). Such factors can often create barriers to vaccination and are further compounded by language barriers, misinformation, misconceptions, reduced literacy, limited access to technology, lack of trust, homelessness, and migrant or refugee statuses. One solution to this problem is comprehensive clinical trials with robust, clear evidence on the potential benefits and risks of exposure to COVID-19 vaccines, giving pregnant women the privilege to make informed decisions regarding their health and the health of their newborns.

Here, we suggest that the COVID-19 vaccine should be tested as a critical prevention strategy for pregnant women and their children in ongoing and future clinical trials. We acknowledged that in the absence of a standard vaccine to serve as the control, the use of placebo may be considered; however, the ethics of administering a potentially ineffective therapy to combat a morbid viral infection is unquestionably harmful. This argument is compounded by the risk posed to both the mother and the fetus. Ultimately, these trials may have lower enrollment given the potential risk of contracting COVID-19 when given the placebo. Nevertheless, we hope these trials will elucidate the efficacy, safety, and long-term outcomes of COVID-19 vaccination in pregnant mothers and the developing fetus. And we strongly suggest monitoring the incidence of maternal fever, acetaminophen use, rate of pregnancy loss, developmental outcomes in the newborn, and ADHD in offspring of pregnant women who have received COVID-19 vaccination. By monitoring the outcomes for pregnant and lactating women and their babies, these investigations will further promote informed decision-making for COVID-19 vaccination during pregnancy and lactation, and may lead to a reduction in vaccine-hesitancy.

As the COVID-19 pandemic continues, a public health obligation exists to evaluate pregnant women and their children (in all populations and ethnic groups) in well-designed and well-funded COVID-19 vaccine trials to identify and implement appropriate prevention and care based on strong evidence.

## Author Contributions

CY and ST conceived the manuscript. CY and HZ conducted the search and drafted the manuscript. CY, HZ, and ST revised the manuscript. All authors contributed to the article and approved the submitted version.

## Funding

This study was supported by the Canadian Institutes of Health Research (CIHR) (Grant No. 177747).

## Conflict of Interest

The authors declare that the research was conducted in the absence of any commercial or financial relationships that could be construed as a potential conflict of interest.

## Publisher’s Note

All claims expressed in this article are solely those of the authors and do not necessarily represent those of their affiliated organizations, or those of the publisher, the editors and the reviewers. Any product that may be evaluated in this article, or claim that may be made by its manufacturer, is not guaranteed or endorsed by the publisher.

## Footnotes

1
*In Vitro* Fertilization; https://www.newlifeivf.com.au/your-fertility-treatment-and-covid-19/

## References

[1] Galang RR Newton SM Woodworth KR Griffin I Oduyebo T Sancken CL. Risk Factors for Illness Severity Among Pregnant Women With Confirmed SARS-CoV-2 Infection-Surveillance for Emerging Threats to Mothers and Babies Network, 22 State, Local, and Territorial Health Departments, March 29, 2020 -March 5, 2021. Clin Infect Dis (2021) 73:S17–23. doi: 10.1093/cid/ciab432 PMC8194562 34021332

[2] Shimabukuro TT Kim SY Myers TR Moro PL Oduyebo T Panagiotakopoulos L. Preliminary Findings of mRNA Covid-19 Vaccine Safety in Pregnant Persons. N Engl J Med (2021) 384:2273–82. doi: 10.1056/NEJMoa2104983 PMC8117969 33882218

[3] Poston L Caleyachetty R Cnattingius S Corvalán C Uauy R Herring S. Preconceptional and Maternal Obesity: Epidemiology and Health Consequences. Lancet Diabetes Endocrinol (2016) 4:1025–36. doi: 10.1016/S2213-8587(16)30217-0 27743975

[4] Cummings MJ Baldwin MR Abrams D Jacobson SD Meyer BJ Balough EM. Epidemiology, Clinical Course, and Outcomes of Critically Ill Adults With COVID-19 in New York City: A Prospective Cohort Study. Lancet (2020) 395):1763–70. doi: 10.1016/S0140-6736(20)31189-2 PMC7237188 32442528

[5] Waller DK Hashmi SS Hoyt AT Duong HT Tinker SC Gallaway MS. Maternal Report of Fever From Cold or Flu During Early Pregnancy and the Risk for Noncardiac Birth Defects, National Birth Defects Prevention Study, 1997-2011. Birth Defects Res (2018) 110:342–51. doi: 10.1002/bdr2.1147 PMC5831519 29094488

[6] Graham JM Jr. Update on the Gestational Effects of Maternal Hyperthermia. Birth Defects Res (2020) 112:943–52. doi: 10.1002/bdr2.1696 32686349

[7] Gou X Wang Y Tang Y Qu Y Tang J Shi J. Association of Maternal Prenatal Acetaminophen Use With the Risk of Attention Deficit/Hyperactivity Disorder in Offspring: A Meta-Analysis. Aust N Z J Psychiatry (2019) 53:195–206. doi: 10.1177/0004867418823276 30654621

[8] Liew Z Ritz B Rebordosa C Lee PC Olsen J. Acetaminophen Use During Pregnancy, Behavioral Problems, and Hyperkinetic Disorders. JAMA Pediatr (2014) 168:313–20. doi: 10.1001/jamapediatrics.2013.4914 24566677

[9] Tiegs G Karimi K Brune K Arck P. New Problems Arising From Old Drugs: Second-Generation Effects of Acetaminophen. Expert Rev Clin Pharmacol (2014) 7:655–62. doi: 10.1586/17512433.2014.944502 25075430

[10] Liew Z Ritz B Virk J Olsen J. Maternal Use of Acetaminophen During Pregnancy and Risk of Autism Spectrum Disorders in Childhood: A Danish National Birth Cohort Study. Autism Res (2016) 9:951–8. doi: 10.1002/aur.1591 26688372

[11] Collier AY McMahan K Yu J Tostanoski LH Aguayo R Ansel J. Immunogenicity of COVID-19 mRNA Vaccines in Pregnant and Lactating Women. JAMA (2021) 325:2370–80. doi: 10.1001/jama.2021.7563 PMC8120446 33983379

[12] Perl SH Uzan-Yulzari A Klainer H Asiskovich L Youngster M Rinott E. SARS-CoV-2-Specific Antibodies in Breast Milk After COVID-19 Vaccination of Breastfeeding Women. JAMA (2021) 325:2013–4. doi: 10.1001/jama.2021.5782 PMC8042567 33843975

[13] PrabhuDas M Adkins B Gans H King C Levy O Ramilo O. Challenges in Infant Immunity: Implications for Responses to Infection and Vaccines. Nat Immunol (2011) 12:189–94. doi: 10.1038/ni0311-189 21321588

[14] Kim L Whitaker M O'Halloran A Kambhampati A Chai SJ Reingold A. Hospitalization Rates and Characteristics of Children Aged <18 Years Hospitalized With Laboratory-Confirmed COVID-19 - COVID-NET, 14 States, March 1-July 25, 2020. MMWR Morb Mortal Wkly Rep (2020) 69:1081–8. doi: 10.15585/mmwr.mm6932e3 PMC7440125 32790664

[15] Snook ML Beigi RH Legro RS Paules CI. Should Women Undergoing In Vitro Fertilization Treatment or Who are in the First Trimester of Pregnancy be Vaccinated Immediately Against COVID-19. Fertil Steril (2021) 116:16–24. doi: 10.1016/j.fertnstert.2021.05.083 34148583 PMC8118645

[16] Malizia BA Hacker MR Penzias AS. Penzias.Cumulative Live-Birth Rates After *In Vitro* Fertilization. N Engl J Med (2009) 360:236–43. doi: 10.1056/NEJMoa0803072 19144939

[17] Chmielewska B Barratt I Townsend R Kalafat E van der Meulen J Gurol-Urganci I. Effects of the COVID-19 Pandemic on Maternal and Perinatal Outcomes: A Systematic Review and Meta-Analysis. Lancet Glob Health (2021) 9:9e759–772. doi: 10.1016/S2214-109X(21)00079-6 PMC8012052 33811827

